# Olfactory neuroblastoma: the long-term outcome and late toxicity of multimodal therapy including radiotherapy based on treatment planning using computed tomography

**DOI:** 10.1186/s13014-015-0397-5

**Published:** 2015-04-15

**Authors:** Takashi Mori, Rikiya Onimaru, Shunsuke Onodera, Kazuhiko Tsuchiya, Koichi Yasuda, Hiromitsu Hatakeyama, Hiroyuki Kobayashi, Shunsuke Terasaka, Akihiro Homma, Hiroki Shirato

**Affiliations:** Department of Radiation Oncology, Hokkaido University Hospital, Sapporo, Japan; Department of Radiation Medicine, Hokkaido University Graduate School of Medicine, North 15 West 7, Kita-ku, Sapporo 060-8638 Japan; Department of Otolaryngology-Head and Neck Surgery, Hokkaido University Graduate School of Medicine, Sapporo, Japan; Department of Neurosurgery, Hokkaido University Graduate School of Medicine, Sapporo, Japan

**Keywords:** Olfactory neuroblastoma, Computed tomography, Three-dimensional conformal radiotherapy, Late adverse effect

## Abstract

**Background:**

Olfactory neuroblastoma (ONB) is a rare tumor originating from olfactory epithelium. Here we retrospectively analyzed the long-term treatment outcomes and toxicity of radiotherapy for ONB patients for whom computed tomography (CT) and three-dimensional treatment planning was conducted to reappraise the role of radiotherapy in the light of recent advanced technology and chemotherapy.

**Methods:**

Seventeen patients with ONB treated between July 1992 and June 2013 were included. Three patients were Kadish stage B and 14 were stage C. All patients were treated with radiotherapy with or without surgery or chemotherapy. The radiation dose was distributed from 50 Gy to 66 Gy except for one patient who received 40 Gy preoperatively.

**Results:**

The median follow-up time was 95 months (range 8–173 months). The 5-year overall survival (OS) and relapse-free survival (RFS) rates were estimated at 88% and 74%, respectively. Five patients with stage C disease had recurrence with the median time to recurrence of 59 months (range 7–115 months). Late adverse events equal to or above Grade 2 in CTCAE v4.03 were observed in three patients.

**Conclusion:**

Multimodal therapy including radiotherapy with precise treatment planning based on CT simulation achieved an excellent local control rate with acceptable toxicity and reasonable overall survival for patients with ONB.

## Introduction

Olfactory neuroblastoma (ONB), or esthesioneuroblastoma, is a rare tumor originating from olfactory epithelium. In a meta-analysis of studies published from 1990 through 2000, the cases of 390 patients were reported in 26 studies, and the averaged overall and disease-free survival rates at 5 years were 45% and 41%, respectively [1]. In ONB patients with metastases in cervical lymph nodes (on average 5% of the total), the survival rate was 29%, compared with 64% for patients with N0 disease (odds ratio 5.1), and a combination of surgery and radiotherapy was suggested to be the optimum approach to treatment [1]. Platek et al. reported that between 1973 and 2006 there were 511 patients in the Surveillance, Epidemiology, and End Results (SEER) database, which collects cancer incidence and survival data from cancer registries that are population-based and cover approx. 26% of the United States population [2]. Those authors showed that the 5-year overall survival stratified by treatment modality was 73% for surgery with radiotherapy, 68% for surgery only, 35% for radiotherapy only, and 26% for neither surgery nor radiotherapy, suggesting that surgery with radiotherapy is the optimal strategy.

However, a standard strategy of treatment for ONB has not been established, and the optimal radiotherapy dose, the exact role of chemotherapy, and the effectiveness of elective neck dissection are yet to be confirmed. Because of the low incidence of ONB, it is difficult to conduct prospective trials to test new treatments. Retrospective studies can still be used to investigate treatment efficacy and toxicities.

In our hospital, precise simulations using computed tomography (CT) and three-dimensional dose calculation have been used in the radiotherapy for all head and neck cancer patients since the 1980s [3,4]. Since 1992, we have also used the clinical target volume (CTV) delineated slice by slice using 5-mm-thick slices from CT images, and patients have been treated with three-dimensional conformal technologies. In the present study, we retrospectively investigated the patients with ONB who were treated with the CT simulation. The purpose of this study was to analyze the relationship between treatment modalities, survival, tumor control, and the acute and late toxicity of patients treated for ONB with radiotherapy with or without surgery or chemotherapy.

## Materials and methods

### Patients

The Institutional Review Board at Hokkaido University Hospital approved this retrospective study in May 2013. Seventeen patients with ONB were treated with definitive intent with radiotherapy at the Department of Radiotherapy, Hokkaido University Hospital between July 1992 and June 2013. We retrospectively reviewed their medical records including the initial diagnosis, tumor extension, treatment modalities and follow-up, and we used demographic information collected up until September 2014. The patient characteristics are presented in Table 1. All patients had a pathological diagnosis.

**Table 1:** **Patient Characteristics and Treatments**

**Patient No.**	**Gender**	**Age**	**Kadish stage**	**LN meta**	**Treatment**	**Surgery**	**Chemotherapy**	**Radiation dose**
1	F	22	B	–	Surgery + PostopRT	TR	–	65Gy/26Fr
2	M	59	B	–	Surgery + PostopRT	PR	–	60Gy/30Fr
3	F	72	B	–	Surgery + PostopRT	STR	–	50Gy/25Fr
4	M	53	C	–	Surgery + PostopRT	STR	–	56Gy/28Fr
5	F	66	C	RPN	Surgery + PostopRT	TR	–	65Gy/26Fr
6	M	63	C	–	PreopRT + Surgery		–	40Gy/20Fr
7	M	55	C	–	NAC + Surgery + PostopRT	STR	ICE 1 cycle	65Gy/26Fr
8	F	71	C	–	NAC + Surgery + PostopRT	TR	ICE 1 cycle	60Gy/30Fr
9	F	43	C	–	NAC + Surgery + PostopRT	TR	ICE 5 cycle	60Gy/30Fr
10	M	37	C	–	NAC + Surgery + PostopRT	TR	ICE 5 cycles	60Gy/30Fr
11	M	66	C	–	NAC + Surgery + PostopRT	TR	CDDP	60Gy/24Fr
12	F	44	C	–	NAC + Surgery + PostopRT	STR	CDDP + ETP	60Gy/30Fr
13	M	46	C	–	Surgery + Chemo + PostopRT	PR	ICE 2 cycles	66Gy/33Fr
14	M	24	C	–	NAC + definitive RT	–	CADO/CVP 3 cycle	54Gy/27Fr
15	F	67	C	ILCN	NAC + definitive RT	–	ICE 2 cycles	66Gy/33Fr
16	M	70	C	–	NAC + definitive RT	–	CDDP 1 cycles + ICE 2 cycles	65Gy/26Fr
17	M	76	C	–	definitive RT + Chemo	–	ICE 2 cycles	65Gy/26Fr

Abbreviations: RPN (retropharyngeal node), ILCN (ipsilateral cervical node), PostopRT (postoperative radiotherapy), PreopRT (preoperative radiotherapy), NAC (neoadjuvant chemotherapy), TR (total resection), STR (subtotal resection), PR (partial resection), ICE (Ifosfamide, cisplatin and etoposide), CDDP (cisplatin), ETP (etoposide), CADO/CVP (cisplatin, cyclophosphamide, vincristine, etoposide and doxorubicin).

The median age of the patients at diagnosis was 59 years (range, 22–76 yrs). Ten of the 17 patients were male and seven were female. According to the Kadish classification, three patients were classified as stage B (paranasal sinus involved) and 14 patients were classified as stage C (extension beyond paranasal sinus). There was no patient with stage A ONB. Lymph node metastases were observed in two patients at initial diagnosis: one patient had retropharyngeal lymph node (RPN) metastasis and the other had ipsilateral cervical node metastasis.

### Treatments

The patients’ treatments are summarized in Table 1. Five patients were treated with surgery and postoperative radiotherapy. One patient was treated with preoperative radiotherapy and surgery. Six patients were treated with neoadjuvant chemotherapy (NAC), surgery, and postoperative radiotherapy. One patient was treated with surgery followed by chemotherapy and sequential postoperative radiotherapy. Three patients were treated with NAC and definitive radiotherapy. One patient was treated with definitive radiotherapy followed by chemotherapy.

### Surgery

We defined ‘total resection’ as no residual tumor after surgery, ‘subtotal resection’ as resected macroscopic tumor but microscopic residual tumor, and ‘partial resection’ as macroscopic residual tumor. Six, four, and two patients underwent total, subtotal, and partial resections, respectively.

Three patients with stage B ONB underwent endoscopic sinus surgery (ESS) (two patients) or tumor resection as Denker’s operation (one patient). One patient with stage B ONB who was treated with ESS and postoperative irradiation underwent a biopsy from the cribriform plate on the day the radiotherapy was completed and residual tumor was observed; skull base surgery was thus performed for this persistent disease.

Ten patients with stage C ONB underwent a radical resection combining both otolaryngologic and neurosurgical resection. No cervical lymph node dissection was performed in any patient.

### Chemotherapy

All three patients with stage B ONB were treated with surgery and postoperative radiotherapy without chemotherapy. Among the 14 patients with stage C disease, three were treated without chemotherapy; the combination of surgery and postoperative radiotherapy was performed for two patients, and preoperative radiotherapy and surgery was performed for one patient.

Six patients with stage C disease were treated with NAC followed by surgery and postoperative radiotherapy. Four of these patients were treated with the ICE (ifosfamide, cisplatin and etoposide) regimen and the other two patients were treated with only cisplatin or cisplatin and etoposide. One patient was treated with surgery and postoperative sequential chemoradiotherapy. In this patient, only the intracranial lesion was resected, followed by postoperative chemotherapy (ICE regimen in two cycles) and postoperative irradiation to cranial and nasal lesions.

Three patients were treated with a combination of NAC and radiotherapy without surgery. All were treated with chemotherapy followed by radiotherapy. The regimen of chemotherapy was ICE in two cycles for one patient, carboplatin in one cycle and ICE in two cycles for one patient and CADO/CVD (cisplatin, cyclophosphamide, vincristine, etoposide and doxorubicin) regimen in three cycles for one patient. The other patient was treated with definitive radiotherapy followed by chemotherapy (ICE regimen in two cycles).

### Radiotherapy

All patients were immobilized with a thermoplastic mask in supine position at the time of simulation and irradiation. The contrast-enhanced or plain planning CT was obtained in five and in twelve patients respectively. The planning CT and MRI fusion was used in four patients. The radiation delivery was three-dimensional conformal radiotherapy (3DCRT) in 16 patients and intensity-modulated radiation therapy (IMRT) in one patient. X-ray energy was 6 MV in 14 patients and 10 MV in two patients. One patient was treated with Co-60 initially and with 10 MV X-ray in boost irradiation. Electron beams with adequate energy were additionally used with the photon beams when the dose to the tumor using X-rays was insufficient to avoid organs at risk such as the eye or brain. Electron beams were typically used for the ethmoid or frontal sinus.

The CTV included primary tumor or tumor bed and postoperative resection cavity in postoperative cases, nasal cavity, ethmoid bone and sphenoid bone in 15 patients treated with 3DCRT. In addition, ipsilateral or bilateral maxillary sinuses were included in four and one patients respectively. One patient with RPN metastasis, who underwent a resection of the primary lesion, was then irradiated only for the RPN with 65 Gy in 26 fractions.

In one patient treated with IMRT, the CTV consisted of primary tumor identified in MRI obtained before and after neoadjuvant chemotherapy, nasal cavity, ethmoid bone, bilateral maxillary sinus and bilateral RPN and upper jugular lymph node (level II) regions.

The fraction size was 2.0 Gy or 2.5 Gy. When 2.5 Gy was used, the irradiation delivery was done 4 times per week. When 2.0 Gy was used, irradiation delivery was done 5 times per week.

Postoperatively, the planned doses were 50 to 66 Gy in 2 Gy or 2.5 Gy per fraction. In one patient with preoperative radiotherapy, the radiation dose was 40 Gy in 20 fractions.

There were three patients treated with sequential chemoradiotherapy. The radiation dose was 65 Gy in 26 fractions after carboplatin in one cycle and ICE in two cycles in one patient. In one patient with CADO/CVD in three cycles, 54 Gy in 27 fractions was used. One patient with ipsilateral cervical node metastasis was treated with ICE in two courses and then irradiated at the primary site with 66 Gy in 33 fractions; the involved cervical lymphatic region was irradiated with 40 Gy in 16 fractions. In addition, a stereotactic boost with 28 Gy in 4 fractions to the sphenoid bone and 10 Gy in 4 fractions to the ethmoid sinus was performed for this patient. The overall treatment time for radiotherapy ranged from 40 to 67 days (median 44 days).The radiotherapy treatment planning systems were a Focus or Xio (Computerized Medical Systems, St. Louis, MO) in ten patients, the CT-THERAC (NEC Corp., Tokyo) in three patients, the Eclipse (Varian Medical Systems, Palo Alto, CA) in two patients for the 3DCRT, and the Pinnacle (Philips Healthcare, Best, The Netherlands) in two patients for the 3DCRT and IMRT.

### Statistical analysis

Adverse effects of radiotherapy were evaluated based on the NCI Common Terminology Criteria for Adverse Events (CTCAE) version 4.03. We retrospectively graded late adverse effects as the most severe one until last available consultation. Overall survival (OS) and relapse-free survival (RFS) were estimated using the Kaplan-Meier method. The period of relapse was defined as the time between the initial diagnosis and relapse or death, whichever occurred first. Statistical analyses were performed using JMP software, version 11.0.0 (SAS Institute, Cary, NC). The p-values lower than 0.05 were considered significant.

## Results

**Figure 1:**
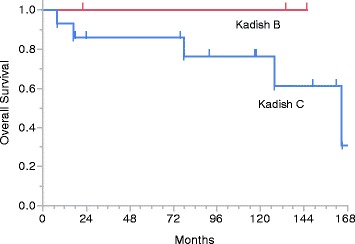
Kaplan-Meier plot of overall survival.

**Figure 2:**
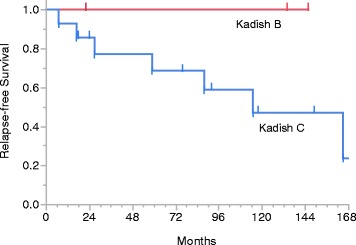
Kaplan-Meier plot of relapse-free survival.

The median follow-up time was 95 months (range 8–173 months). The 5-year OS was 88% (95% confidence interval [CI] 63.2% − 97.0%) and the 5-year RFS was 74% (95%CI 46.6% − 89.9%). There was no significant difference between the Kadish stage B and C patients in OS (p = 0.303, Figure 1) or RFS (p = 0.214, Figure 2).

Five patients, all of whom had stage C disease, had recurrence. The median time to recurrence was 59 months (range 7–115 months). The pattern of recurrence was intracranial recurrence in two patients, ipsilateral cervical node metastasis in two patients, and spinal dissemination in one patient. All of them were out of radiation field or distant metastasis. The patient who suffered from spinal dissemination died 1 month after the diagnosis of dissemination. This patient received NAC following IMRT. One patient with ipsilateral cervical node metastasis died 18 months after the diagnosis of recurrence without no salvage treatment. This patient was treated with definitive radiotherapy followed by adjuvant chemotherapy. The other three patients survived more than 24 months (24, 47 and 101 months, respectively) from the time of recurrence by salvage treatments such as surgical resection or radiotherapy. Ten patients (eight of whom were treated with surgery and two who were not) had no relapse. For two other patients, it is unknown whether they had disease or not with follow-up periods of 17 and 146 months.

**Figure 3:**
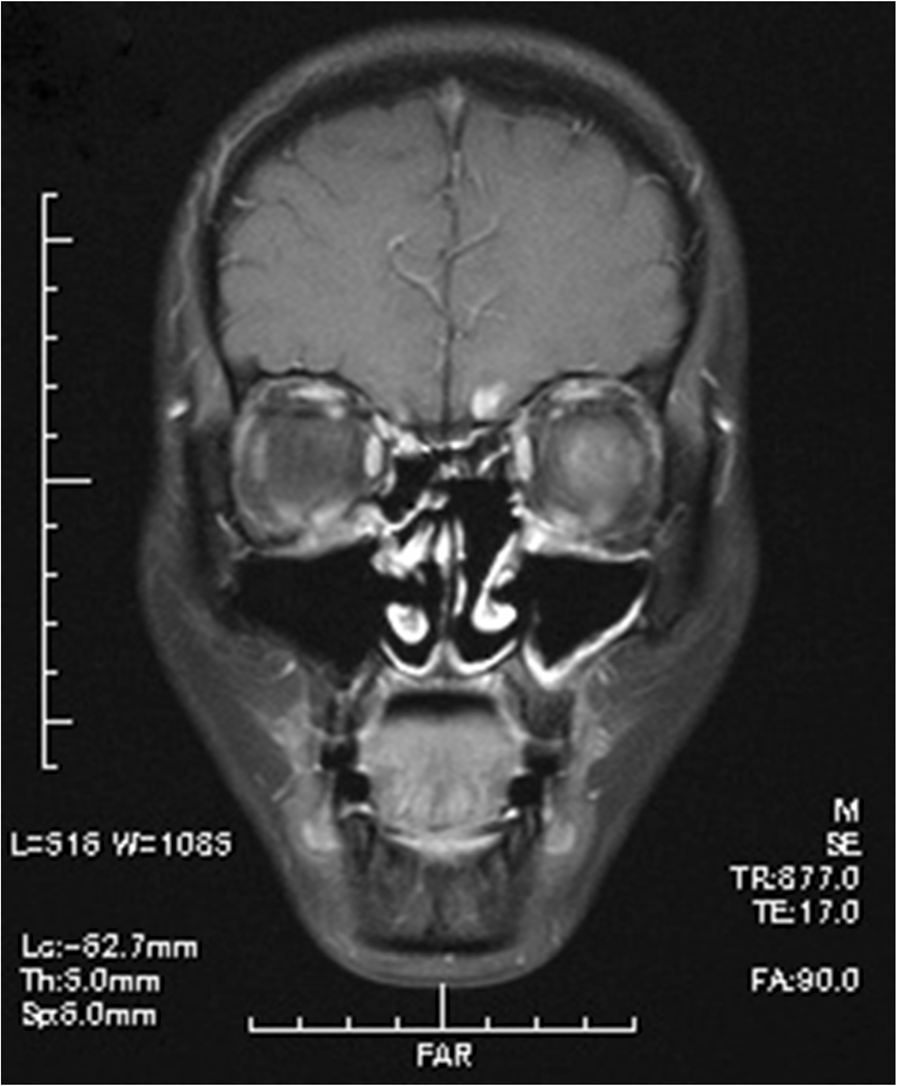
Coronal view of follow-up MRI 10 years after radiotherapy (patient 1). Focal enhancement at the base of the frontal lobe was observed. This finding disappeared in follow-up MRI after 9 months.

Acute adverse effects such as radiation dermatitis, mucositis of nasal mucosa, and conjunctivitis were within Grade 1–2 and tolerable in all patients. One patient was affected with intracranial epidural abscess, when radiotherapy was performed till 36 Gy and radiotherapy was suspended during 13 days. One patient died 81 days after the completion of radiotherapy and one patient was followed up in another institution after the completion of radiotherapy, so 15 out of the 17 patients were eligible for long-term toxicities evaluation. Late adverse effects were observed in three patients. Three patients showed abnormal findings thought to be radiation injury at the frontal and left temporal lobes, the frontal and bilateral temporal lobes, and the left frontal lobe in follow-up brain MRI at 23, 47 and 57 months after radiotherapy, respectively, but they showed no symptoms (Figure 3).

**Figure 4:**
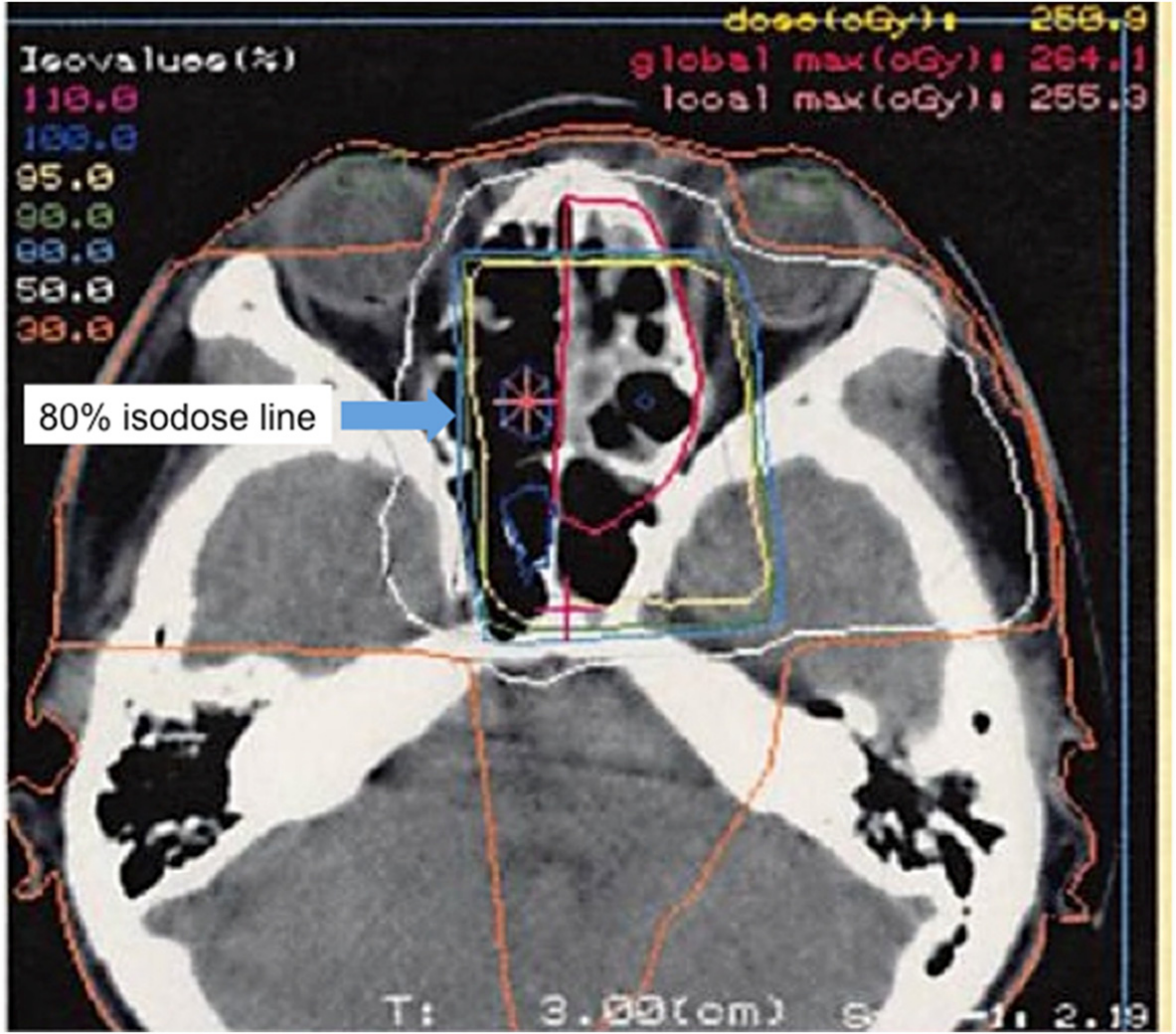
Dose distribution at the level of orbit (patient 1).

**Figure 5:**
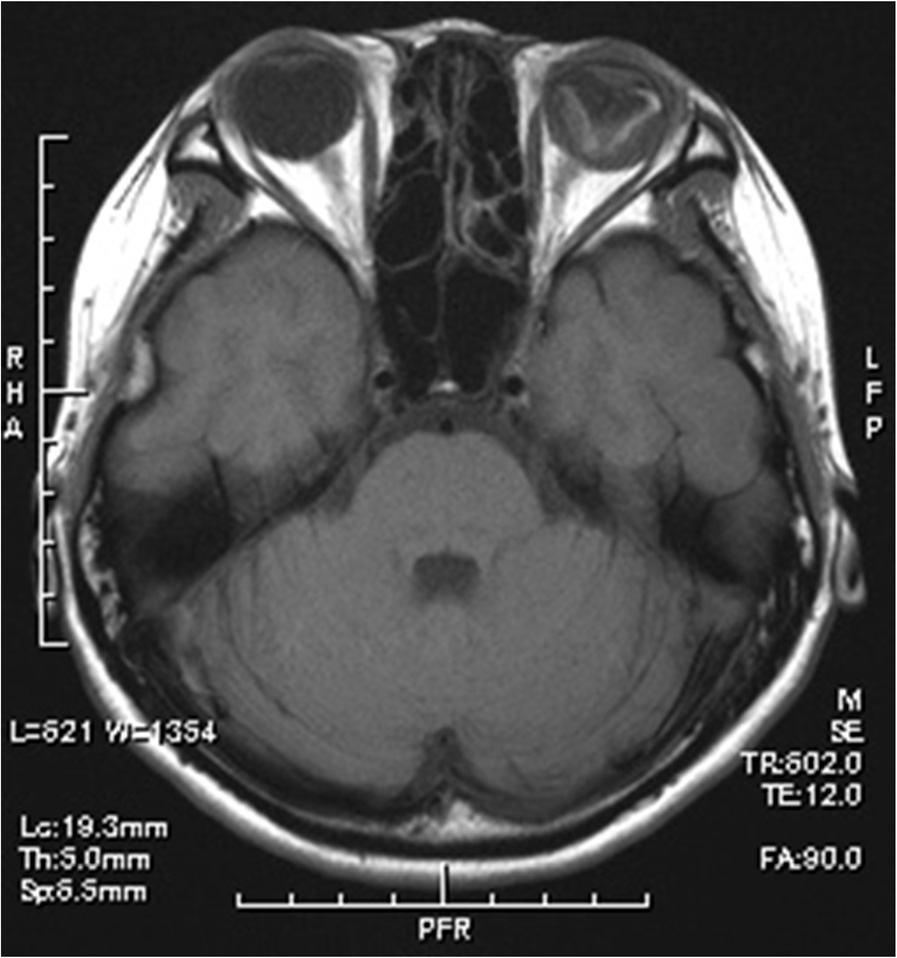
Axial view of follow-up MRI 10 years after radiotherapy (patient 1). Retinopathy of the left eye was observed.

**Figure 6:**
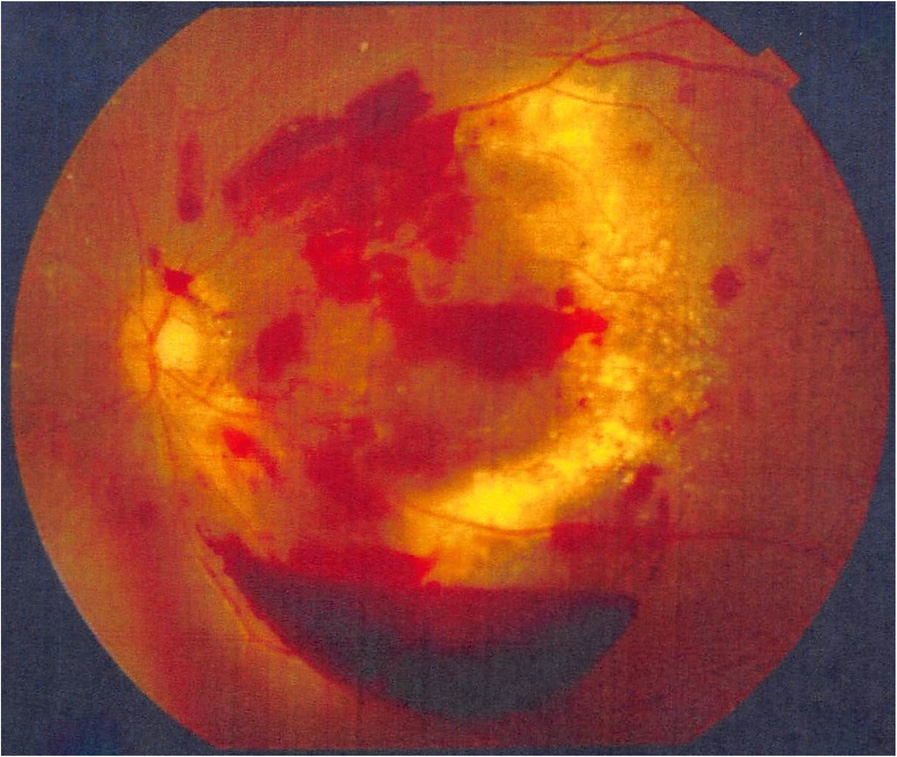
Fundus photograph of the left eye 10 years after radiotherapy (patient 1). Exudate and vitreous hemorrhage are observed.

Late adverse effects equal to or above Grade 2 in CTCAE v4.03 were observed in all of these patients; two patients showed Grade 2 hypopituitarism such as hypothyroidism or adrenal insufficiency at 75 and 103 months after the completion of radiotherapy. One of these patients was prescribed 66Gy in 33 fractions with a stereotactic boost to the ethmoid sinus with 10 Gy in four fractions and to the sphenoid bone with 28 Gy in four fractions (patient 13). The other patient was prescribed 65 Gy in 26 fractions (patient 1), and the dose to the pituitary sinus was estimated as 52 to 62 Gy in 26 fractions from the dose distribution. This patient also suffered from Grade 4 glaucoma, retinopathy, and vitreous hemorrhage and needed ophthalmological intervention. The maximum dose to the eye was estimated as 58 Gy in 26 fractions from the dose distribution (Figure 4). This patient treated with X-ray and electron beam, and the radiation injury occurred in the area that matched the field of X-ray and electron beam. A follow-up MRI and a fundus photograph of the patient’s left eye 10 years after the radiotherapy are shown in Figure 5 and Figure 6.

The other patient suffered from the necrosis of frontal bone 33 months after complete of radiotherapy and needed necrotic bone removal (patient 9). She was prescribed 60 Gy in 30 fractions. The necrotic part of frontal bone was included in radiation fields and maximum dose of it was about 62 Gy in 30 fractions from the dose volume histogram. No hot spot above 62 Gy within frontal bone was observed in the dose distribution.

## Discussion

Eich et al. reported that the 5-year OS was 64% in an ONB population of 14 stage B patients and 33 stage C patients with multimodality therapy including surgery, radiotherapy, and chemotherapy [5]. Ozsahin et al. reported that the OS at 5 years was 52% (95% CI 34%–70%) in stage C patients with multimodality therapy including surgery, radiotherapy and chemotherapy [6]. In the present study, the OS and RFS at 5 years were 88% and 74%, respectively. Grade 2 or above late radiation toxicities occurred in three patients. These OS and RFS values are comparable to those of previous reports and tend to be higher than previously reported outcomes (Table 2). Our cohort ranged over more than 20 years and anti-cancer treatment has dramatically changed throughout these periods: surgical technique improvements and extended use of chemotherapy may have also modified the patient outcomes. Though the outcomes of radiotherapy based on treatment planning using CT with multimodal therapy are encouraging, it is difficult to come to the conclusion that it is only due the role of the 3DCRT.

**Table 2:** **Studies reporting treatment outcomes for ONB**

**Author**	**Year**	**Period**	**Patients (n)**	**Treatment**	**Follow up (mo)**	**5-year OS**	**Other Survival (yr)**
Gruber [14]	2002	1980-2001	28	Surgery + Pre-/PostopRT or RT +/− CTX	68	77%	DFS 70% (5 yr)
Eich [5]	2003	1979-2001	47	Surgery only, Surgery + PostopRT, RT +/− CTX or multimodality therapy	65	64%	EFS 50% (5 yr)
Diaz [7]	2005	1979-2002	30	Surgery only, Surgery + Pre-/PostopRT or RT +/− CTX	87	89%	RFS 69% (5 yr)
Ozsahin [6]	2010	1971-2004	77	Surgery only, Surgery + RT, RT +/− CTX or multimodality therapy	72	64%	DFS 57% (5 yr)
Platek [2]	2011	1973-2006	511	Surgery only, Surgery + RT, RT only or neither surgery nor RT	–	73% Surgery + RT,	–
						68% Surgery only,	
						35% RT only,	
						26% neither surgery nor RT	
Modesto [9]	2013	1998-2010	43	multimodality therapy	77	65%	PFS 57% (5 yr)
Present study	–	1992-2013	17	Surgery + PostopRT, PreopRT + Surgery or multimodality therapy	95	88%	RFS 74% (5 yr)

Abbreviations: RT (radiotherapy), PostopRT (postoperative radiotherapy), CTX (chemotherapy), DFS (disease-free survival), EFS (event-free survival), RFS (relapse-free survival), PFS (progression-free survival).

We used 50 to 66 Gy as postoperative radiotherapy, 40 Gy as preoperative radiotherapy, and 54 to 66 Gy in the setting of sequential chemoradiation therapy. Diaz et al. reported a mean radiation dose of 56.9 Gy (range 50.0 − 67.2 Gy) in a postoperative setting [7]. Bacher et al. reported the mean dose of radiation as 53.13 Gy (median 50 Gy, range 50–60 Gy) for the preoperative setting and 54.57 Gy (median 55 Gy, range 45–60 Gy) for the postoperative setting [8]. Other studies described postoperative doses ranging from 55 to 65 Gy, preoperative doses at 50 Gy, and inoperative patients receiving up to 70 Gy [9,1]. Because of the rarity of ONB, it is still difficult to establish the optimal radiation dose.

In our study, eleven of the 17 patients received chemotherapy, and nine of these eleven patients received NAC. Kim et al. reported that two of 11 patients with ONB who received NAC consistent with etoposide, ifosfamide and cisplatin showed complete responses and seven of 11 patients showed partial responses [10]. Mishima et al. reported the results of NAC and radiotherapy with or without peripheral blood stem cell support or surgery [11]. They reported that eight of 12 patients with ONB showed complete responses after the treatment.

Noh et al. reported that failure in a cervical node did not occur in their ONB patients who were treated with systemic chemotherapy without elective cervical nodal irradiation [12]. However, Elkon et al. reported the results of surgery and radiotherapy without chemotherapy in which the 5-year OS was 75%, 60% and 41% for Kadish stage A, B and C patients, respectively [13]. Gruber et al. reported that the DFS at 5 years was 70% without chemotherapy [14]. Despite the small number of patients in each study and some biases including technical advancement, the results of the treatment with chemotherapy seem to be better than those achieved without chemotherapy. Our present findings do not contradict the hypothesis that the addition of chemotherapy may improve survival in patients with ONB treated with radiotherapy.

Although elective nodal irradiation was not used in the present patient series, only two patients experienced ipsilateral cervical node metastases (27 and 59 months after radiotherapy). One patient (patient 5) was initially diagnosed with RPN metastasis and only the primary lesion was resected. She then underwent irradiation only for the RPN with 65 Gy in 26 fractions without chemotherapy. The other patient (patient 17) was treated with definitive radiotherapy with 65 Gy in 26 fractions followed by chemotherapy. Some investigators have proposed the necessity of elective nodal irradiation. Demiroz et al. reported that elective nodal failure occurred in seven of 26 patients and recommended the use of elective nodal irradiation [15]. Only two patients received chemotherapy in that study. However, Noh et al. reported that no cervical failure was observed in nine patients treated with a multimodal approach including systemic chemotherapy. They concluded omitting elective nodal may be an option when patients are treated with a combination of radiotherapy and chemotherapy [12]. In our study, there was one patient with cervical nodal recurrence among the eleven patients who received chemotherapy. Although the small number of patients makes it difficult to make any definite conclusions, we cannot exclude the potential role of elective nodal irradiation in the eradication of microscopic lymph node metastasis even when systemic chemotherapy is used.

Glaucoma, retinopathy and vitreous hemorrhage occurred together in one patient in our study. This patient was treated with 3DCRT using X-rays and electron beam. It is possible that these late toxicities resulted from the uncertainty in field matching. In order to reduce these late toxicities further in this patient, IMRT or proton beam therapy (PBT) may have been helpful. One of our patients (patient 12) received IMRT, and he experienced spinal dissemination and died 81 days after the completion of radiotherapy, and thus any late toxicities of this patient could not be evaluated. The efficacy of IMRT for tumor in nasal cavity and paranasal sinuses has been reported [16,17]. In a subset analysis of patients with tumors of the paranasal sinuses and nasal cavity treated with IMRT in a recent investigation by Wiegner et al., the 2-year local regional control rate and the 2-year OS of ONB were 86% and 100% respectively. They also reported that six (11.5%) out of 52 patients had late toxicity equal to or above Grade 3 including corneal ulcer in one patient [16]. Dirix et al. retrospectively compared toxicities between groups treated with IMRT and those treated with 3DCRT postoperatively for tumors of the paranasal sinuses or nasal cavity. They found no radiation-induced visual impairment nor Grade 3 or 4 late toxicity in 39 patients who received IMRT whereas six (15.8%) radiation-induced retinopathy resulting in mild to moderate visual impairment in 38 patients in the 3DCRT group [17].

PBT may also contribute to a reduction of late toxicities and may help reduce the risk of secondary malignancy compared to IMRT. Nishimura et al. reported the preliminary results of PBT: no Grade 3 or above late toxicities were observed other than skin reaction, and one patient with Kadish C experienced liquorrhea after tumor shrinkage [18]. Nichols et al. and Herr et al. also reported results of multimodality therapy including PBT [19,20]. They observed nine Grade 3 complications and one case of Grade 4 radiation-induced optic neuritis with an average follow-up of 73 months. Technical advances in radiation oncology such as IMRT and PBT can result in less toxicity in the treatment of ONB.

Several investigators reported that recurrence occurs long after the completion of ONB treatment. Loy et al. reported the mean relapse interval of 6 years [21]. Gruber et al. reported that five of 28 patients had recurrence over 60 months after the completion of treatment [14]. Bacher et al. reported that the median time from diagnosis to recurrence was 57 months [8]. In our study, the median follow-up time was 95 months (range 8–173 months). Although it was not possible to identify the actual cause of death in three of the patients in our series, the follow-up time was one of the longest in the literature. Long-term follow-ups are necessary for the evaluation of ONB treatments in prospective settings.

In this study, we retrospectively reviewed medical records. Some kinds of adverse effects, especially symptomatic or low grade ones, can be missed if they were not written in medical records. Therefore, we may underestimate the incidence of adverse effects of radiotherapy. In addition, the small number of patients without control group and treatment heterogeneity makes interpretation of results very difficult.

Nowadays 3DCRT is very common treatment technique, but because of low incidence of ONB, retrospective studies as this present study using 3DCRT can still be meaningful to investigate treatment efficacy and toxicities of ONB and we can expect the improvement of treatment outcome and less toxicity hereafter.

In conclusion, it is suggested that multimodal therapy including radiotherapy with precise treatment planning based on CT simulation was effective for ONB in terms of local control and overall survival. More advanced radiation technique such as IMRT and PBT providing more optimal dose delivery can reduce late toxicities and improve treatment outcome of ONB.
